# Association between the Degree of Processing of Consumed Foods and Sleep Quality in Adolescents

**DOI:** 10.3390/nu12020462

**Published:** 2020-02-12

**Authors:** Raíssa da Silva Sousa, Maylla Luanna Barbosa Martins Bragança, Bianca Rodrigues de Oliveira, Carla Cristine Nascimento da Silva Coelho, Antônio Augusto Moura da Silva

**Affiliations:** 1 Departamento de Ciências Fisiológicas, Universidade Federal do Maranhão, São Luís, Maranhão 65080-040, Brazil; sousasraissa@gmail.com; 2 Programa de Pós-Graduação em Saúde Coletiva, Universidade Federal do Maranhão, São Luís, Maranhão 65020-070, Brazil; oliveirarodrigues00@gmail.com (B.R.d.O.); carlacristinecoelho@gmail.com (C.C.N.d.S.C.); aamouradasilva@gmail.com (A.A.M.d.S.)

**Keywords:** food consumption, ultra-processed foods, sleep, adolescents

## Abstract

The aim of this study was to evaluate the association between food consumption by the degree of processing and sleep quality in adolescents from São Luís, Maranhão, Brazil. A cross-sectional study with 2499 adolescents (aged 18 to 19 years) was developed. Exposure variables included energy contributions of food groups stratified by the NOVA classification: fresh or minimally processed foods (FMPF), processed foods (PF), and ultra-processed foods (UPF), categorized into quartiles. The outcome variable was sleep quality assessed with the Pittsburgh Sleep Quality Index. Associations between these variables were estimated by Poisson regression, with robust estimation of variance. Most of the adolescents had poor sleep quality (57.1%). There were associations between FMPF in the third (57.1%–66.0% of total calories; prevalence ratio PR = 0.88; 95% CI: 0.80, 0.97) and fourth quartile (66.1%–95.8% of total calories; PR = 0.87; 95% CI: 0.78, 0.96) and lower prevalence of poor sleep quality. The fourth quartile of UPF (44.3%–81.8% of total calories; PR = 1.14; 95% CI: 1.03, 1.27) was associated with a higher prevalence of poor sleep quality. Higher intake of FMPF is a protective factor for poor sleep quality, whereas higher UPF consumption is a risk factor for poor sleep quality.

## 1. Introduction

Lifestyle changes of the past few decades have increasingly led to changes in food consumption [1], such as the reduced consumption of fresh or minimally processed foods (FMPF) and increased consumption of ultra-processed foods (UPF) [2]. In this context, Brazilian epidemiologists developed the NOVA food classification based on observations that the increase in epidemics of obesity and type II diabetes was associated with an increase in UPF consumption [3]. This classification was created according to the degree of processing, dividing foods into four groups: FMPF, processed culinary ingredients, processed foods (PF), and UPF [4].

According to the NOVA classification, fresh foods are obtained directly from plants or animals without undergoing any changes. Minimally processed foods correspond to fresh foods that have been subjected to cleaning processes, removing inedible or undesirable parts. Culinary ingredients are those extracted directly from fresh foods and are consumed as items of culinary preparations. This group of foods is rarely consumed in the absence of FMPF. Processed foods are those produced with the addition of salt, sugar or another substance for culinary use to increase durability and improve its sensory qualities. In addition, they are recognized as versions of the original foods even after processing. Unlike this group, ultra-processed foods do not preserve their basic identity when submitted to processing steps, they are formulated by industry, synthesized in the laboratory, made entirely or mostly of substances extracted from food, in addition to being nutritionally unbalanced [4].

Studies based on the NOVA classification have shown that the replacement of minimally processed foods with ultra-processed products is associated with an unhealthy diet and various non-communicable diseases [3]. In addition, evidence indicates that eating high-fat and sugar-rich foods and excess calories daily may be associated with poor sleep quality [5]. However, there are no studies of the association between food consumption by the degree of processing and sleep quality.

Sleep is considered an essential factor for learning and memory and acts directly on hormonal and behavioral regulation. Therefore, sleep has a significant role in the development of children and adolescents, that are in a stage of intense learning [6]. Sleep quality is directly linked to quality of life, because inadequate sleep can affect the full development of adolescents, thereby causing harm to psychosocial health, compromising school performance, and even fostering the development of risk behaviors [7].

Eating habits and sleep are important factors to be considered in relation to adolescent health. Therefore, it is essential to evaluate the relationship between these two parameters [8]. Moreover, there was an increase in UPF consumption [1] and problems related to sleep quality in adolescents [7,9,10] suggesting the possibility of a deleterious effect of UPF consumption on sleep quality. To the best of our knowledge, there are no published reports of research that has studied this possible association. Therefore, this study was conducted to evaluate the association between the degree of processing of foods in the diet and sleep quality in adolescents.

## 2. Materials and Methods

This study protocol was approved by the Research Ethics Committee of the University Hospital of the Federal University of Maranhão (approval no. 1302,489 on 29 October 2015). All participants provided written informed consent.

This cross-sectional analysis was conducted on data obtained from a study of a birth cohort in São Luís, Maranhão, Brazil, titled “Determinants along the life cycle of obesity, precursors of chronic diseases, human capital and mental health.”

This cohort included hospital births of mothers living in the city of São Luís, from March 1997 to February 1998, and involved 10 public and private hospitals. Systematic sampling with stratification proportional to the number of births in each hospital was used. Thus, one in seven deliveries per hospital were recruited. In this initial phase, a total of 2542 births were included. After excluding stillbirths, the study sample comprised 2443 births [11].

This cohort underwent a new evaluation as adolescents (aged 18 to 19 years) from January to December 2016. To locate participants, we searched school and university enrollment, addresses and contacts, on social media, as well as military enrollment records (for males). A total of 654 adolescents were identified and agreed to participate in this follow-up study. Due to the difficulty in locating individuals, we non-randomly enrolled other adolescents born in the municipality of São Luís in 1997 to participate in the birth cohort study to increase the power of the sample and to prevent future statistical losses. Strategies used to identify new participants included school, university, and military enrollment registration (for males). To this end, individuals were included in two ways: by lot from the “*Sistema de Informação sobre Nascidos Vivos*” (National Live Births Registry, SINASC in the Portuguese acronym) database, and by the inclusion of volunteers identified in schools and universities who born in a maternity from São Luís in 1997. From this listing, the random draw was made giving 4593 individuals born in 1997 in São Luís. Of this total, we contacted 1716 people by telephone or personal contact. In a second stage, volunteers born in the same year were identified in schools, universities and social media. Thus, an additional 1861 adolescents were included in the survey. The new participants were submitted to the same tests and questionnaires as the others of the original cohort. A total of 2515 adolescents were enrolled. However, 16 participants were excluded because of missing data on sleep quality or food intake. Therefore, data from 2499 participants were considered for analysis in this study.

Data were collected by trained health professionals and recorded in the online program Research Electronic Data Capture (Redcap^®^) [12]. Data on sociodemographic, lifestyle, and anthropometric characteristics were obtained by standardized questionnaires. The following variables were studied: sex (male and female), age (in years), education (elementary, middle, technical or vocational course, and college), economic class (A, B, C, and DE, with class A being the most affluent) according to the criteria of the *Associação Brasileira de Empresas de Pesquisa* (Brazilian Association of Research Enterprises, ABEP in the Portuguese acronym) [13], separated or divorced parents (yes and no), and screen time per day (≤ 5 h and > 5 h), frequency of coffee consumption (daily, weekly, monthly, never) and amount of coffee consumed in cups (180 mL) per day (< 0.1, 0.1 to 1.0, > 1.0).

Food information was obtained through a food frequency questionnaire (FFQ) and investigated for the last 12 months before the interview. This instrument was developed by Schneider et al. [14] and was adapted to the food intake of adolescents from São Luís, Maranhão. The FFQ had 106 food items, and the usual average frequency of consumption of the listed foods was obtained with the daily, weekly, or monthly consumption time unit. Eight response options were used for frequency of consumption: never or < 1 time/month; 1–3 times/month; 1 time/week; 2–4 times/week; 5–6 times/week; 1 time/day; 2–4 times/day; and ≥ 5 times/day [15].

The average portion-size photos of each food were made available for computer viewing to minimize memory bias and improve the accuracy of portion-size information. It was recorded whether the adolescent consumed the food in the visualized portions (medium portion), greater quantity (large portion), or smaller quantity (small portion). The average portion of the food in grams or milliliters was obtained from the Table for Assessment of Food Consumption in Homemade Measures [16]. From the median portion, we obtained values in grams or milliliters for the large portion (1.5 of the median portion) and small portion (0.5 of the median portion).

To estimate the energy consumption of food, the frequency of consumption reported in each item was converted to annual consumption in order to capture less frequent consumption. Never or <1 time/month was considered as absent consumption; 1–3 times/month has been transformed to 12.00 times/year; 1 time/week was transformed to 52.00 times/year; 2–4 times/week has been transformed to 104.00 times/year; 5–6 times/week has been transformed to 260.00 times/years; 1 time/day has been transformed to 365.25 times/year; 2–4 times/day has been transformed to 730.50 times/year; ≥5 times/day was transformed to 1826.25 times/year. Subsequently, the annual frequency was converted to daily frequency then divided by 365.25. Daily food intake in grams or milliliters was calculated by multiplying the daily frequency by the portion size that was recorded.

Nutrient intake was obtained from the knowledge of macronutrient values in 100 g or mL of each food or preparation. For this, we used the *Tabela Brasileira de Composição de Alimentos* (Brazilian Table of Food Composition, TACO in the Portuguese acronym) [17] and the *USDA Nutrient Database for Standard Reference* [18], or food labeling information. The energy intake of each food was estimated by multiplying the carbohydrate and protein values by 4 kcal and lipid values by 9 kcal. The daily energy consumption of each food was obtained on the basis of the total calories from each macronutrient. Total daily energy intake was assessed by adding all the calories consumed from all FFQ items.

Sleep quality was assessed using the Pittsburgh Sleep Quality Index (PSQI), validated in Brazil by Bertolazi et al. [19]. The PSQI questionnaire was prepared by Buysse et al., [20] and focus on evaluate sleep quality over the last month. In Brazil, this instrument was validated for adolescents by Passos et al. [21]. The scale has quantitative and qualitative sleep information to estimate sleep quality. The sleep quality questionnaire consists of 19 self-administered questions and five questions answered by the roommates of the subject. These roommates answers were used for clinical information only and were not included in this study. The 19 questions have a weighted distribution on a scale from 0 to 3. The PSQI assesses the following sleep-related components: subjective sleep quality, sleep latency, sleep duration, habitual sleep efficiency, sleep disorders, use of medication for sleep, and daytime dysfunction. The scores of the components are summed to produce an overall score ranging from 0 to 21. Thus, scores from 0 to 4 indicated good sleep quality, and scores above 4 implied poor sleep quality.

A theoretical causality model based on a directed acyclic graph (DAG) was elaborated. In this graphic, we included the exposure, outcome, and confounding variables. Causal connections represented by arrows were established among all variables (Supplementary Figure S1). Each variable in the DAG is represented by a rectangle and the colors have different meanings: the variable in green and with the “►” symbol inside the rectangle was the exposure variable; that in blue and with the letter “I” inside the rectangle was the response variable; variables in blue are the antecedents of the outcome variable; and those in red are antecedents of the outcome and exposure variables, which are variables selected to compose the minimum set for confounding adjustment. For the elaboration of the graphical model, we used the online software Dagitty, version 3.2 [22].

After the construction of the graphical model, a minimum set of confounding variables was selected by the backdoor criterion [23], which were used to adjust the analysis, in order to avoid unnecessary adjustments, spurious associations, and estimation errors. The models were adjusted by the following minimum set of variables: age, education, economic class, marital status of parents, and screen time.

The data were analyzed using the STATA^®^ statistical software, version 14.0. Descriptive analysis of the studied variables was conducted. Categorical variables were described by absolute and relative frequencies, and continuous variables were described as means and standard deviations. The amount of daily intake of nutrients from the UPF group was compared to the fraction of nutrients from the FMPF group and the nutrients from the PF. For these comparisons, we used the Student’s *t*-test.

To verify the association between food consumption and sleep quality, we conducted a Poisson regression analysis with robust variance estimation because it is a cross-sectional study with a high prevalence of the dependent variable [24]. Unadjusted and adjusted analysis for the variables indicated by the DAG were conducted. The level of significance was set at 0.05. Interactions between sex and energy contributions of the studied groups were tested, and no significant differences were detected; therefore, we included participants of both sexes in the same model. For the interaction analysis, the significance level was established at *p* < 0.10.

## 3. Results

The study population predominantly comprised adolescents aged 18 years (69.1%), females (52.3%), those with high school education (67.1%), belonging to economic class B (48.6%), with separated or divorced parents (51.5%), with screen time of up to 5 h per day (66.0%), who consumed coffee daily (55.1%), with coffee ingestion of up to one serving daily (52.6%). When comparing these characteristics in relation to poor and good sleep quality, we found that female adolescents (62.7%), students in elementary school (84.0%) and those aged 19 years (60.2%) had a higher percentage of poor sleep quality (Table 1). Most adolescents presented poor sleep quality (57.1%) (data not shown in table).

Adolescents with poor sleep quality had lower average energy contributions from FMPF (56.1%), higher energy contribution from UPF (36.4%) and higher consumption of added sugars (72.2 g/day) (Table 2).

The average energy contributions for the adolescents studied were 56.9% from FMPF, 4.6% from PF and 35.8% from UPF. Adolescents from the last quartile presented the highest energy contributions for each food group. Thus, the FMPF contributed to 73.5% of the diet, PF to 10.6%, and UPF to 53.0% of overall energy (Table 3).

An evaluation of the association between food consumption according to the degree of processing and sleep quality found that, relative to the first quartile, the second (48.1 to 57.0% of total calories; prevalence ratio PR = 0.89;95% CI: 0.82, 0.98), third (57.1 to 66.0% of total calories; PR = 0.89; 95% CI: 0.81, 0.97), and fourth (66.1 to 95.8% of total calories; PR = 0.88; 95% CI: 0.80, 0.96) FMPF energy contribution quartiles were associated with a lower prevalence of poor sleep quality. After adjusting for the confounding variables (education, economic class, marital status of parents, age, and screen time), the associations persisted for the third (PR = 0.88; 95% CI: 0.80, 0.97) and fourth (PR = 0.87; 95% CI: 0.78, 0.96; Table 4) quartiles.

In relation to the energy contribution of PF, we found an association between the second quartile and a higher prevalence of poor sleep quality (1.7 to 3.2% of total calories; PR = 1.11; 95% CI: 1.01, 1.22). This association was not maintained after adjustment for confounding variables (PR = 1.09; 95% CI: 0.98, 1.21). Regarding the energy contribution of UPF, we observed an association between the second (26.3 to 35.0% of total calories; PR = 1.10; 95% CI: 1.00, 1.22) and fourth quartiles (44.3%–81.8% of total calories; PR = 1.13; 95% CI: 1.02, 1.24) with the highest prevalence of poor sleep quality. After adjusting for confounding variables, the association persisted only for the fourth quartile (PR = 1.14; 95% CI: 1.03; 1.27; Table 4).

## 4. Discussion

To our knowledge, this is the first study that evaluated the association between food intake, categorized according to the NOVA classification for processed foods, and sleep quality. Our main findings were that the greatest energetic contribution of the adolescents’ diet came from the FMPF, however most of the adolescents had poor sleep quality. Those with poor sleep quality had lower energy contributions from FMPF, higher energy contribution from UPF and higher consumption of added sugar. The higher energy contributions from FMPF were associated with a lower prevalence of poor sleep quality, whereas high energy contributions from UPF were associated with a higher prevalence of poor sleep quality. The results of this study can contribute to the development of other studies that assess causality between food consumption according to the degree of processing and sleep quality.

An earlier population-based study by Louzada et al. [25] in subjects who were 10 years of age or older followed the same classification for food and used data from the *Pesquisa de Orçamentos Familiares no Brasil* (Family Budget Research, POF in the Portuguese acronym; 2008–2009) [1], found high energy contribution from FMPF (69.5%), followed by the UPF (21.5%), and the PF (9.0%). In comparison, in our results, we observed a higher energy contribution from UPF than the mentioned study. This finding is worrisome because high consumption of foods with a high degree of processing is associated with metabolic syndrome in adolescents [26].

Ultra-processed products have increased their participation in the Brazilian diet since 1980 in metropolitan areas and throughout the country since the 2000 s [27]. About 40% of Brazilian teenagers consume at least one of the following ultra-processed foods daily: sweets, candies, chocolates, chewing gum, chocolates or lollipops, soft drinks and salted processed/ultra-processed foods, such as hamburger, ham, bologna, salami, sausage, sausage, instant noodles, packaged snacks and crackers [28]. In this study, we found that adolescents with poor sleep quality had lower energy contribution from FMPF, higher energy contribution from UPF and higher consumption of added sugar. This result was expected once UPFs are rich in added sugars [4] and this nutrient has been associated with worse quality [29] and short sleep [30] in adolescents and young adults.

In relation to the sleep quality of the evaluated adolescents, we found that most had poor sleep quality. This finding is similar to that of Hoefelmann et al. [31] who found 45.7% of 14–24-year-olds had poor sleep quality. Explanations for this high prevalence may be related to biological, social, and behavioral factors such as age, economic class, physical inactivity, screen time, school activities, caffeine intake, and chronic diseases [9,10,32,33,34,35].Despite the considerable coffee consumption among the studied adolescents and evidence that high and moderate caffeine consumption is associated with sleep disorders in adolescents [36,37], there was no significant difference in coffee consumption between the groups of good and poor sleepers.

We did not found studies that addressed the association between food consumption according to the degree of processing and sleep quality. Our study showed that adolescents who consumed more FMPF had a lower prevalence of poor sleep quality. A potential mechanism by which FMPF can positively influence sleep quality is that the group comprises nutrient-rich foods that can attenuate symptoms of poor sleep quality [38]. Some of these nutrients are: tryptophan, isoflavones, selenium, vitamin C, vitamin D and fiber [39,40,41,42]. One of the nutrients of FMPF is tryptophan – an amino acid that directly affects sleep regulation and increases brain serotonin production (which reduces sleep latency) [39]. Isoflavones, which are bioactive compounds that act as antioxidants, have estrogen-mimetic action, and affect serotonergic function and regulate the sleep–wake cycle [40]. Selenium and vitamin C perform immunoregulatory function, preventing the exacerbation of immune responses that can lead to chronic inflammation and poor sleep quality [41,42]; vitamin D reduces difficulty in maintaining sleep and fibers are associated with less deep sleep [38,43]. Similarly, in a cross-sectional study with Japanese women aged 34 to 65 years, a positive association was observed between sleep quality and high intake of vegetables and fish [44]. Our results tend to that direction because adolescents with lower prevalence of poor sleep quality had the highest energy contribution from FMPF and higher intake of antioxidants, proteins, fiber, vitamins, and minerals such as selenium and vitamin C.

In this study, we observed that adolescents who consumed more UPF had a higher prevalence of poor sleep quality. These associations may occur due to the nutritional composition of these foods [1,16,17,18]. In a sleep intervention study, St-Onge et al. [43] found that higher saturated fat and carbohydrate intake was associated with lighter sleep profile and more nocturnal sleep arousals, respectively. In a systematic review, people who slept poorly were observed to have a diet with a higher caloric contribution of carbohydrates and fats when compared to those who slept well [45]. We observed a high carbohydrate consumption among our subjects with a lower prevalence of poor sleep quality; however, this consumption was associated with a higher intake of fiber, vitamins, and minerals, and thereby justifies the positive impact of this macronutrient on sleep quality.

High UPF consumption contributes to being overweight [46]. In line with our results, a study of overweight children and adolescents between 10 and 16 years of age found that overweight participants had shorter sleep duration and more sleep disturbances compared to eutrophic individuals. From this perspective, overweight adolescents are more likely to have poor sleep quality [47]. These data indicate one of the possible mechanisms by which UPF would negatively interfere with sleep quality through a domino effect. Furthermore, the higher consumption of UPF is related to an increased inflammatory response, either due to the presence of chemical additives or to the dietary composition of the foods [48,49,50]. Inflammatory markers have a significant role in various clinical conditions [51,52]. For example, Irwin et al. [53], in a systematic review and meta-analysis, observed a positive association between sleep disturbances and increased levels of C-reactive Protein (CRP) and interleukin-6 (IL-6)—two important inflammatory markers. Nonetheless, it is noteworthy that poor sleep quality may increase consumption of UPF, due to greater neural stimuli resulting from sleep restriction, which further leads individuals to consume high amounts of UPF [54]. As a consequence, the consumption of UPF may worsen health quality, as they contribute to overweight and obesity, as well as cause hormonal, metabolic, and physiological changes that lead to worse sleep quality [43,46,47]. However, as this is a cross-sectional study, it was not possible to assess whether the consumption of AUP causes bad sleep.

The limitations of this study include memory bias when answering questions on food consumption. To minimize this bias, interviewers were trained to assure the proper application of the questionnaire. In addition, we need to highlight that the FFQ can overestimate food consumption. However, it is an instrument based on the principle that the estimation of the habitual diet represents a more important exposure factor than punctual consumption [55], and therefore it is preferable in studies that assess the intensity of exposure [56]. One factor that contributes to this overestimation is the way items are grouped in the FFQ because of the difficulty in separating some foods according to the type of processing (e.g., white bread, which can be processed or ultra-processed). To minimize a potential bias caused by this situation, in each of these cases, we assigned the food to the category of NOVA classification in which it is most commonly used. Therefore, white bread was classified as a processed food. Another study limitation involves selection bias caused by the inclusion of a subsample of 1861 adolescents in the last phase of the study. However, we found that there was no difference between the group of adolescents from the birth phase compared to the group that was included in the adolescence phase for the main subject characteristics of economic class, skin color, marital status of parents, and nutritional status of the adolescent. One of the strengths of this study is the use of the NOVA food classification, which groups foods according to their nature, purpose, and extent of industrial processing and not merely by nutrients and type of food [4]. Another strength of this study is the use of the Pittsburgh Scale, which is validated for use to assess sleep quality in adolescents [21].

## 5. Conclusions

Most of the adolescents studied had poor sleep quality, with a considerable percentage of energy contribution from FMPF and UPF. Higher energy contributions from FMPF were associated with lower prevalence of poor sleep quality, whereas high energy contributions from UPF were linked with a higher prevalence of poor sleep quality. Considering that adolescence is a window of opportunity for building and attaining habits that tend to endure into adulthood, it is worthwhile to modulate life habits based on the results of this study. FMPF intake is a protective factor for good sleep quality while UPF intake is a risk factor for poor sleep quality. Therefore, sleep quality needs to be more frequently addressed as an indicator of public policies that focus to develop intersectoral action guidelines which can stimulate the adoption of practices associated with good sleep quality, such as increased FMPF intake and reduced UPF intake. The results of this study can contribute to the development of other studies that assess causality between food consumption according to the degree of processing and sleep quality.

## Figures and Tables

**Table 1:** Sociodemographic and lifestyle characteristics according to sleep quality of adolescents, São Luís, Maranhão, Brazil, 2016.

Variables	Good Sleep Quality	Poor Sleep Quality	Total	*p*-Value
	% (*n*)	% (*n*)	% (*n*)	
Age (years)				0.037
18	44.2 (764)	55.8 (963)	69.1 (1727)	
19	39.8 (307)	60.2 (465)	30.9 (772)	
Sex				<0.001
Male	48.9 (584)	51.1 (609)	47.7 (1193)	
Female	37.3 (487)	62.7 (819)	52.3 (1306)	
Schooling				0.030
Elementary	16.0 (4)	84.0 (21)	1.0 (25)	
Medium	43.6 (730)	56.4 (946)	67.1 (1676)	
Technical/vocational course	46.5 (60)	53.5 (69)	5.2 (129)	
College	41.4 (277)	58.6 (392)	26.7 (669)	
Economic class ^1,2^				0.136
A	45.4 (78)	54.6 (94)	7.8 (172)	
B	43.9 (473)	56.1 (605)	48.6 (1078)	
C	39.9 (371)	60.1 (559)	42.0 (930)	
D/E	52.8 (19)	47.2 (17)	1.6 (36)	
Separated/Divorced Parents				0.262
Yes	43.9 (565)	56.1 (721)	51.5 (1286)	
No	41.7 (506)	58.3 (707)	48.5 (1213)	
Screen time				0.542
≤5 h per day	42.4 (700)	57.6 (950)	66.0 (1650)	
>5 h per day	43.7 (371)	56.3 (478)	34.0 (849)	
Frequency of coffee consumption				0.654
Daily	43.2 (595)	56.8 (782)	55.1 (1377)	
Weekly	41.1 (218)	58.9 (313)	21.3 (531)	
Monthly	49.1 (26)	50.9 (27)	2.1 (53)	
Never	43.1 (232)	56.9 (306)	21.5 (538)	
Amount of coffee consumed (cup/day)				0.978
<0.1	43.1 (232)	56.9 (306)	21.5 (538)	
0.1 to 1.0	42.7 (561)	57.3 (754)	52.6 (1315)	
>1.0	43.0 (278)	57.0 (368)	25.9 (646)	

^1^ Economic class of 2216 adolescents. ^2^ Average family income per month in each economic class in 2016: A = US$ 5989, B = US$ 2021, C = US$ 1241, D/E = US$ 220.

**Table 2:** Daily nutrient intake according to sleep quality in adolescents from São Luís, Maranhão, Brazil, 2016.

Daily Nutrient Intake	Good Sleep Quality (*n* = 1071)		Poor Sleep Quality (*n* = 1428)	
	Mean	SD	Mean	SD
FMPF Energy contribution (%) ^1^	57.9	12.9	56.1	13.2
PF Energy contribution (%)	4.5	4.4	4.6	4.5
UPF Energy contribution (%) ^1^	34.9	13.1	36.4	13.2
Calories (kcal)	2999.9	1369.0	2971.2	1409.4
Carbohydrates (g)	468.5	214.3	460.9	215.3
Protein (g)	103.5	50.0	101.5	49.3
Total fat (g)	79.1	44.8	80.1	48.0
Polyunsaturated fatty acids (g)	13.8	8.3	13.6	8.5
Monounsaturated fatty acids (g)	23.7	14.0	24.0	14.6
Saturated fatty acids (g)	29.4	16.8	30.2	18.5
Cholesterol (mg)	422.2	286.1	422.8	284.9
Fibers (g)	38.8	20.1	37.3	19.7
Added sugars (g) ^1^	67.0	54.8	72.2	63.7
Sodium (mg)	2062.8	1135.4	2087.2	1240.0
Selenium (µg)	104.5	49.3	101.3	48.2
Vitamin C (mg)	165.2	152.5	158.1	144.1
Vitamin D (µg)	0.80	0.67	0.82	0.68

^1^*p* < 0.05.

**Table 3:** Energy contribution of separate food groups stratified by the NOVA classification of adolescents from São Luís, Maranhão, Brazil, 2016

Food Groups	Fresh or Minimally Processed Food (FMPF)		Processed Food (PF)		Ultra-Processed Food (UPF)	
Energy Contribution	Mean (%)	SD	Mean (%)	SD	Mean (%)	SD
Total	56.9	13.1	4.6	4.5	35.8	13.1
Quartile 1	39.9	6.6	0.9	0.5	19.5	5.3
Quartile 2	52.8	2.6	2.5	0.4	31.0	2.5
Quartile 3	61.4	2.5	4.3	0.7	39.6	2.6
Quartile 4	73.5	5.6	10.6	5.0	53.0	7.2

SD: standard deviation.

**Table 4:** Association between consumption of minimally processed, processed, and ultra-processed foods and sleep quality in adolescents from São Luís, Maranhão, Brazil, 2016.

Food Groups			Unadjusted Analysis			Adjusted Analysis		
Quartile	*n*	% Minimum and Maximum Values	PR	95% CI	*p*-Value	PR	95% CI	*p*-Value
Fresh or minimally processed								
1°	625	15.1–48.0	Ref.	-	-	Ref.	-	-
2°	625	48.1–57.0	0.89	0.82;0.98	0.025	0.91	0.83;1.00	0.078
3°	625	57.1–66.0	0.89	0.81;0.97	0.016	0.88	0.80;0.97	0.017
4°	624	66.1–95.8	0.88	0.80;0.96	0.008	0.87	0.78;0.96	0.008
**Processed**								
1°	625	0–1.7	Ref.	-	-	Ref.	-	-
2°	625	1.7–3.2	1.11	1.01;1.22	0.030	1.09	0.98;1.21	0.080
3°	625	3.3 - 5.9	1.07	0.97;1.18	0.155	1.07	0.96;1.18	0.199
4°	624	6.0–36.8	1.04	0.94;1.14	0.441	1.04	0.93;1.15	0.432
**Ultra-Processed**								
1°	625	1.2–26.2	Ref.	-	-	Ref.	-	-
2°	625	26.3–35.0	1.10	1.00;1.22	0.047	1.09	0.98;1.21	0.088
3°	625	35.1–44.2	1.08	0.98;1.20	0.100	1.08	0.97;1.20	0.117
4°	624	44.3–81.8	1.13	1.02;1.24	0.011	1.14	1.03;1.27	0.008

PR: prevalence ratio; CI: confidence interval.

## Supplementary Materials

The following are available online at https://www.mdpi.com/2072-6643/12/2/462/s1, Figure S1: Directed acyclic graph (GAD) on food consumption according to processing level and sleep quality in adolescents.
