# Supplementary material for: Association between the Degree of Processing of Consumed Foods and Sleep Quality in Adolescents
Source: Nutrients. 2020 Feb 12;12(2):462. doi: 10.3390/nu12020462 (PMC7071336; doi:10.3390/nu12020462)
Supplement: Supplementary file 1 [file nutrients-12-00462-s001.pdf]

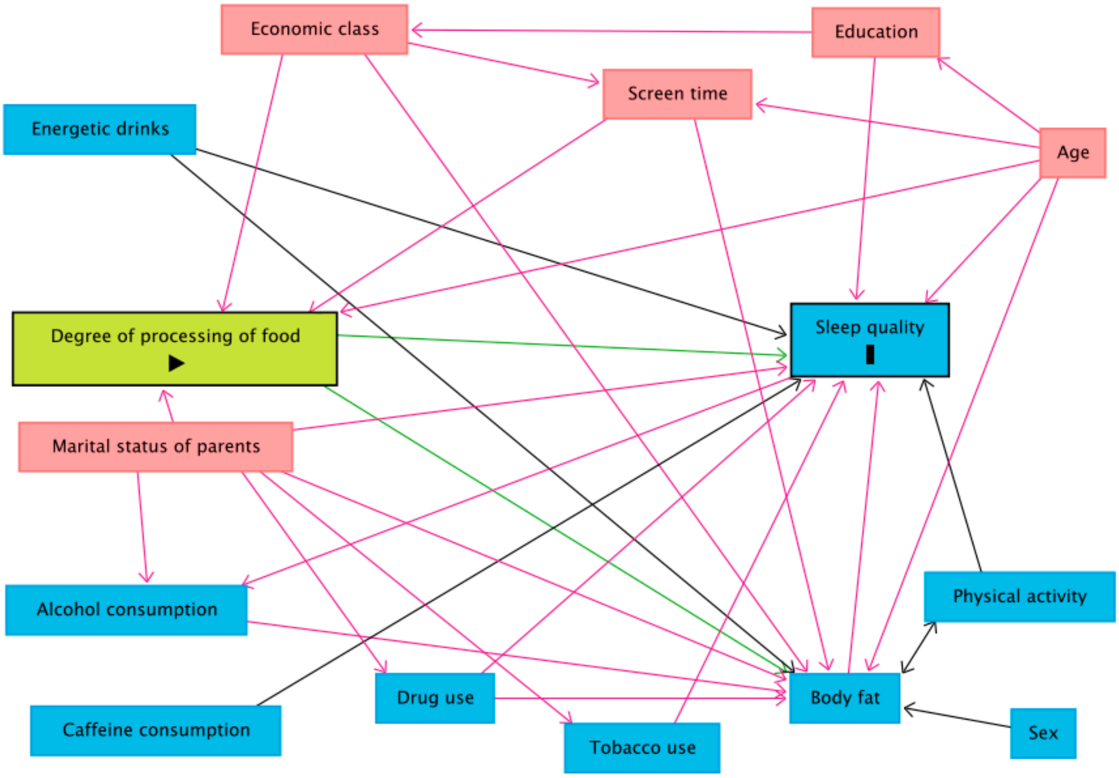

Figure 1. Directed acyclic graph (GAD) on food consumption according to processing level and sleep quality in adolescents.

Legend: The variable in green and with the "►" symbol inside the rectangle was the exposure variable; those in blue and with the letter "I" inside the rectangle were the response variables; variables in blue are the antecedents of the outcome variable; and those in red are antecedents of the outcome and exposure variables.
